# The Theoretical Limits of Watermark Spread Spectrum Sequence

**DOI:** 10.1155/2014/432740

**Published:** 2014-03-23

**Authors:** Nan Jiang, Jian Wang

**Affiliations:** ^1^College of Computer, Beijing University of Technology, Beijing 100124, China; ^2^School of Computer and Information Technology, Beijing Jiaotong University, Beijing 100044, China

## Abstract

At present, the spread spectrum (SS) sequences used in watermark include i.i.d. random sequences and the sequences used in SS communications. They appear earlier than digital watermark. Almost no researchers pay attention to whether they are really fit for watermark. In this paper, we compare the SS watermark channel and the traditional SS communication channel. We find out that their correlation property is different. Considering cropping and translation attacks, we define watermark auto- and cross-correlation and propose Loose Autocorrelation and Tight Cross-Correlation (LAC&TCC) properties for SS watermark. The LAC&TCC properties are that, whether or not synchronized, the autocorrelation is equal or close to 1 and the cross-correlation is equal or close to 0. Accordingly, the peak correlation is divided into the peak autocorrelation *R*_*a*_(*l*) and the peak cross-correlation *R*_*c*_(*l*). We establish the lower bound of *R*_*c*_(*l*) and the higher bound of *R*_*a*_(*l*), respectively. The two bounds indicate that, no matter how small the cover is reserved, the extractor can always find a threshold to distinguish auto- and cross-correlation in theory.

## 1. Introduction

Digital watermarking has been applied to protect digital media from illegal copying and reproduction. Among various watermarking methods [1, 2], spread spectrum (SS) watermarking, originally proposed by Cox et al. [3], is a useful approach. The SS watermarking is developed from SS communications. The watermark is spread over very many frequency bins so that the energy in any one bin is very small and certainly undetectable [3].

By comparing to SS communications whose most key factor is the pseudorandom (PN) sequences, we propose that there are two key components to SS watermarking: the insertion strategy and the PN sequences. However, researchers pay much less attention to the PN sequences than the insertion strategy. At present, the PN sequences, or called PN codes, used in SS watermarking, can be generally categorized into three kinds: independent and identically distributed (i.i.d.) random sequences, the sequences used in SS communications, and other PN-like sequences.

The i.i.d. Gaussian sequences *N*(*μ*, *σ* ^2^) (where *μ* is the mean and *σ*_2_ is the variance) are the most widely used i.i.d. random sequences in SS watermark. Cox et al. first use real valued sequences *N*(0,1) [3]. Since then, many researchers follow the sequences [4–6]. In [7], Kuribayashi and Kato quantize the *N*(0, *σ* ^2^) variable to an integer. Their detailed analysis reveals that the attenuation of the signal energy strongly depends on the quantization performed during the embedding and averaging stages. References [8–10] use the randomly generated sequences taking values from {−1, + 1} with equal probability that can be regarded as the quantization of *N*(0, *σ* ^2^) which quantizes the values that are less than 0 to −1 and greater than 0 to +1.

The SS communications generally make use of a sequential noise-like signal structure, that is, the SS sequences, to spread the normally narrowband information signal over a relatively wideband of frequencies. The receiver correlates the received signals to retrieve the original information signal. The typical sequences that are used in SS communications include m-sequences [11], Gold sequences [12, 13], M-sequences [14], and Walsh sequences [15, 16]. Most of them are used in SS watermarking.

In other PN-like sequences, chaos is the most commonly used. Chaos is a deterministic phenomenon having almost all the features of a noise. The watermark signal generated by chaos system can be embedded in the host image as a small noise-like signal [17, 18]. In addition, because chaos is sensitive to initial conditions, it plays an important role in the watermarking security.

Although all the preceding PN sequences play some role in the SS watermarking, they appear earlier than digital watermarking. They are not specifically designed for watermarking. Almost no researchers pay attention to whether they are really fit for digital watermarking. Only a few papers discuss SS watermarking from the perspective of SS sequences.

Kojima et al. [19] propose a digital watermarking scheme based on complete complementary codes and extend it to steganography. It is shown that the method has superiority to the watermarking scheme based on other SS sequences. The complete complementary codes (pairs of sequence sets) have ideal auto- and cross-correlation properties. It improves the robustness for the collusion attacks.

Huang et al. [20] develop a long PN code based direct sequence spread spectrum (DSSS) flow marking technique for invisibly tracing suspect anonymous flows. One segment of the long PN code is used to spread only one bit of the signal. Benefits of using long PN code include the following: (i) the approach can defeat mean-square autocorrelation based detection technique and make the traceback hard to detect; (ii) it can trace multiple traffic flows in an anonymous network simultaneously.

Deng and Jiang [21] use wavelet sequence as SS codes. Haar wavelet basis is changed into a multilevel orthogonal sequence by scaling and translation, which is used to encode the binary image. Experimental results show that, compared with Hadamard sequence and improved gold sequence, the wavelet multilevel sequence performs better than binary sequence in attack resistance.

In this paper, we discuss the influence of cropping over the correlation of SS sequence in SS watermarking. The peak cross-correlation and the peak autocorrelation are separated and redefined. Based on these definitions, we give the theoretical limits.

## 2. Preliminaries

### 2.1. Watermark Correlations

The correlation properties of sets of SS sequences are important in SS communications. Traditionally the delay or shift receives most attention in auto- and cross-correlation. However, only the delay cannot describe the watermark channel correctly. In watermark channel, attackers use variety of attacks to remove or to render the watermark useless. These attacks can be roughly grouped into signal processing attacks and geometric attacks. In SS watermarking, geometric attacks are difficult to deal with as they involve displacement of pixels, thereby inducing synchronization errors between the original and extracted SS sequences during detection process.

**Figure 1:**
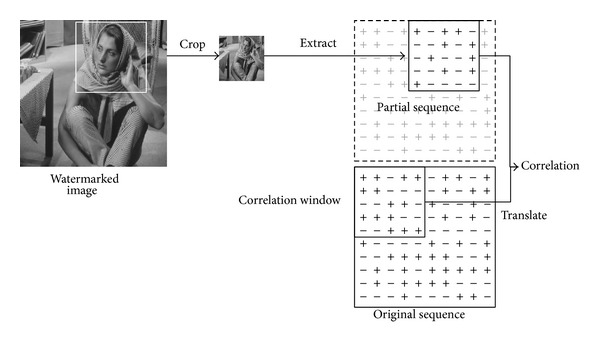
Cropping and translation.

In this paper, we consider cropping and translation because cropping is an easily operable and intractable attack and it is often accompanied by translation. For example, as shown in Figure 1, a user can easily use almost any image processing software to crop an interest part down from a watermarked picture. The extractor can only get partial SS sequence from the cropped picture. We define the size of the partial sequence as correlation window. The extractor calculates the correlation between the original sequence in correlation window and the partial sequence. Because it is difficult to locate the partial SS sequence in the whole original sequence, the correlation is with delay (i.e., translation).

Let *a* = (*a*_0_, *a*_1_,…, *a*_*L*−1_) and *b* = (*b*_0_,  *b*_1_,…,  *b*_*L*−1_) be two bipolar {−1, +1} vectors of length *L*. Then the correlation between *a* and *b* is defined to be the number of positions in which *a* and *b* agree minus the number of positions in which they disagree.

**Definition 1**

Suppose *k* + *l* + *τ* < *L*, the watermark cross-correlation between *a* and *b* over the subsequence of length *l* beginning at position *k* and with relative shift *τ*, denoted by *R*_*ab*_(*τ*, *k*, *l*), is defined by

(1) $$\begin{array}{c} R_{ab}\left(\tau ,k,l\right)=\frac{1}{l}\overset{k+l-1}{\underset{i=k}{\sum }}a_{i}b_{i+\tau }, \end{array}$$

where *k* and *l* are, respectively, the starting position and the size of correlation window and *τ* is the delay.

Similarly, we define the autocorrelation of *a* over *l* consecutive bits beginning at position *k* and with relative shift *τ* by

(2) $$\begin{array}{c} R_{aa}\left(\tau ,k,l\right)=\frac{1}{l}\overset{k+l-1}{\underset{i=k}{\sum }}a_{i}a_{i+\tau }. \end{array}$$

Of course, for any *k* and *l*, we have *R*_*aa*_(0, *k*, *l*) = 1.

### 2.2. Peak Watermark Correlations

In order to improve the anti-interference ability, the traditional SS communications are interested in the maximum peak absolute values of autocorrelations and cross-correlations:

(3) $$\begin{array}{c} R_{\max}=\max\left\{\max\left\{\left|R_{ab}\right|\right\},\max\left\{\left|R_{aa}\right|\right\}\right\}. \end{array}$$

This maximum peak absolute value is the smaller the better. That is to say that the cross-correlation is close to 0 and the autocorrelation is sharp.

However, the property that “the autocorrelation is sharp” is not fit for watermarking. Since the extractor depends on the value of correlation to extract watermarks, the autocorrelation should always be large no matter what attacks the covers suffered. Hence, we present LAC&TCC properties:

- Loose Autocorrelation (LAC): whether or not synchronized, the autocorrelation is equal to or close to 1;
- Tight Cross-Correlation (TCC): whether or not synchronized, the cross-correlation is equal to or close to 0.

Correspondingly, we are interested in the maximum peak absolute values of cross-correlations and the minimum peak absolute values of autocorrelations.

For bipolar sequences *a* and *b*, we define the maximum peak cross-correlation between *a* and *b* over subsequences of length *l* by

(4) Rc(l)=max⁡{|Rab(τ,k,l)|:0≤τ,  k<L,  a≠b}

and the minimum peak autocorrelation of *a* over subsequences of length *l* by

(5) $$\begin{array}{c} R_{a}\left(l\right)=\min\left\{\left|R_{aa}\left(\tau ,k,l\right)\right|:0\leq \tau ,\;k<L\right\}. \end{array}$$

For watermarking, *R*_*c*_(*l*) is the smaller the better and *R*_*a*_(*l*) is the larger the better. The larger *R*_*a*_(*l*) − *R*_*c*_(*l*), the easier to extract watermark and the lower the bit error rate (BER).

## 3. The Theoretical Limits

Usually, it is difficult to give the extract value of *R*_*c*_(*l*) and *R*_*a*_(*l*). But we can give the lower bounds and upper bounds. In the traditional SS communications, there are Welch bound [22, 23], Sarwate bound [24], Sidelnikov bound [25], and so forth. They give the lower bounds of *R*_max⁡_ on how small the cross-correlation and autocorrelation can simultaneously be. In SS watermarking, because we define *R*_*c*_(*l*) and *R*_*a*_(*l*), we should give the lower bound of *R*_*c*_(*l*) and higher bound of *R*_*a*_(*l*), respectively.

### 3.1. The Lower Bound of *R*_*c*_(*l*)

As described above, researchers use different ways to give different lower bounds of *R*_max⁡_. The lower bound of *R*_*c*_(*l*) is a bit similar to that of *R*_max⁡_. Their difference is that *R*_*c*_(*l*) only includes cross-correlation.

**Theorem 2**

In SS watermarking, let Λ be a set of *M* LAC&TCC sequences of length *L* and let *p* be a positive integer; then for every *l* with 1 ≤ *l* ≤ *L*,

(6) $$\begin{array}{c} {\left[R_{c}(l)\right]}^{2p}\geq \frac{1}{M-1}\left[\frac{M}{\left(\begin{array}{c} l+p-1\\ p \end{array}\right)}-1\right]. \end{array}$$

**Proof**

Obviously

(7) $$\begin{array}{c} M\left(M-1\right){\left[R_{c}\left(l\right)\right]}^{2p}\geq \underset{a\neq b}{\sum }{\left[R_{ab}\left(\tau ,k,l\right)\right]}^{2p}=C_{p}. \end{array}$$

We have

(8) Cp=∑a∑b≠a(1l∑i=kk+l−1aibi+τ)2p=1l2p∑a∑b≠a∑i1,i2,…,ip=kj1,j2,…,jp=kk+l−1∏q=1paiqbiq+τajqbjq+τ.

Interchanging orders of summation produces

(9) Cp=1l2p∑i1,i2,…,ip=kj1,j2,…,jp=kk+l−1(∑a∑b≠a  ∏q=1paiqbiq+τajqbjq+τ)=1l2p∑i1,i2,…,ip=kj1,j2,…,jp=kk+l−1((∑a∏q=1paiqbiq+τ)2  −∑a∏q=1paiqaiq+τajqajq+τ).

Because the maximum value of ∏_*q*=1_^*p*^ *a*_*i*_*q*__ *a*_*i*_*q*_+*τ*_ *a*_*j*_*q*__ *a*_*j*_*q*_+*τ*_ is 1, ∑_*a*_∏_*q*=1_^*p*^ *a*_*i*_*q*__ *a*_*i*_*q*_+*τ*_ *a*_*j*_*q*__ *a*_*j*_*q*_+*τ*_ ≤ *M*. So

(10) Cp≥1l2p∑i1,i2,…,ip=kj1,j2,…,jp=kk+l−1((∑a∏q=1paiqbiq+τ)2−M)=1l2p∑i1,i2,…,ip=kj1,j2,…,jp=kk+l−1(∑a∏q=1paiqbiq+τ)2−M.

Various choices of *i*_1_, *i*_2_,…, *i*_*p*_ and *j*_1_, *j*_2_,…, *j*_*p*_ give rise to the same product of *a* and *b*. The number of choices can be expressed as multinomial coefficients. This observation can be used to rearrange the sum in the form

(11) $$\begin{array}{c} C_{p}\geq \frac{1}{l^{2p}}\underset{\begin{array}{c} s_{1},s_{2},\ldots ,s_{l}\\ t_{1},t_{2},\ldots ,t_{l} \end{array}}{\sum }\left(\begin{array}{c} p\\ s \end{array}\right)\left(\begin{array}{c} p\\ t \end{array}\right){\left(\underset{a}{\sum }\overset{l}{\underset{i=1}{\prod }}{\left(a_{i}\right)}^{s_{i}}{\left(b_{i+\tau }\right)}^{t_{i}}\right)}^{2}-M, \end{array}$$

where *s*_*i*_, *t*_*i*_ ≥ 0, ∑_*i*=1_^*l*^ *s*_*i*_ = ∑_*i*=1_^*l*^ *t*_*i*_ = *p*, and

(12) $$\begin{array}{c} \left(\begin{array}{c} p\\ s \end{array}\right)=\frac{p!}{\prod_{i}s_{i}!},\;\;\left(\begin{array}{c} p\\ t \end{array}\right)=\frac{p!}{\prod_{i}t_{i}!}. \end{array}$$

Since the summands are nonnegative, the terms with

(13) $$\begin{array}{c} \left(s_{1},s_{2},\ldots ,s_{l}\right)\neq \left(t_{1},t_{2},\ldots ,t_{l}\right) \end{array}$$

may be dropped to yield

(14) $$\begin{array}{c} C_{p}\geq \frac{1}{l^{2p}}\underset{s_{1},s_{2},\ldots ,s_{l}}{\sum }{\left[\left(\begin{array}{c} p\\ s \end{array}\right)\underset{a}{\sum }\overset{l}{\underset{i=1}{\prod }}{\left(a_{i}\right)}^{2s_{i}}\right]}^{2}-M. \end{array}$$

Cauchy's inequality for sums of squares then gives

(15) $$\begin{array}{c} C_{p}\geq \frac{1}{l^{2p}}\frac{{\left[\sum_{s_{1},s_{2},\ldots ,s_{l}}\left(\begin{array}{c} p\\ s \end{array}\right)\sum_{a}\prod_{i=1}^{l}{\left(a_{i}\right)}^{2s_{i}}\right]}^{2}}{\sum_{s_{1},s_{2},\ldots ,s_{l}}1}-M. \end{array}$$

Interchanging orders of summation and applying the multinomial expansion theorem give

(16) $$\begin{array}{c} C_{p}\geq \frac{1}{l^{2p}}\frac{{\left[\sum_{a}{\left(\sum_{i=1}^{l}a_{i}^{2}\right)}^{p}\right]}^{2}}{\left(\begin{array}{c} l+p-1\\ p \end{array}\right)}-M=\frac{M^{2}}{\left(\begin{array}{c} l+p-1\\ p \end{array}\right)}-M. \end{array}$$

From the previous inequality this yields

(17) $$\begin{array}{c} M\left(M-1\right){\left[R_{c}\left(l\right)\right]}^{2p}\geq \frac{M^{2}}{\left(\begin{array}{c} l+p-1\\ p \end{array}\right)}-M, \end{array}$$

and the theorem follows.

Particularly, when *p* = 1, we have

(18) $$\begin{array}{c} R_{c}\left(l\right)\;\geq \sqrt{\frac{M-l}{1\left(M-1\right)}}. \end{array}$$

### 3.2. The Higher Bound of *R*_*a*_(*l*)

**Theorem 3**

In SS watermarking, let Λ be a set of *M* LAC&TCC sequences of length *L* and let *p* be a positive integer; then for every *l* with 1 ≤ *l* ≤ *L*,

(19) $$\begin{array}{c} R_{a}\left(l\right)\leq \sqrt{\frac{\left(l+2\right)\left(7l+4\right)}{12l^{2}}}. \end{array}$$

**Proof**

Assume that, in *R*_*aa*_(*τ*, *k*, *l*) = (1/*l*)∑_*i*=*k*_^*k*+*l*−1^ *a*_*i*_ *a*_*i*+*τ*_, the number of positions in which *a*_*i*_ and *a*_*i*+*τ*_ agree is *t*; then

(20) $$\begin{array}{c} R_{aa}\left(\tau ,k,l\right)=\frac{1}{l}\left(t-\left(l-t\right)\right)=\frac{1}{l}\left(2t-l\right). \end{array}$$

In order to reduce the BER, *R*_*aa*_(*τ*, *k*, *l*) should be between 0.5 and 1. From 1/2 < *R*_*aa*_(*τ*, *k*, *l*) ≤ 1, we can derive (3/4)*l* < *t* ≤ *l*.

Obviously

(21) $$\begin{array}{c} \overset{l}{\underset{t=(3/4)l+1}{\sum }}{\left[R_{a}(l)\right]}^{2}=\frac{1}{4}l{\left[R_{a}(l)\right]}^{2}\leq \overset{l}{\underset{t=(3/4)l+1}{\sum }}{\left[\frac{1}{l}(2t-l)\right]}^{2}=A_{1}. \end{array}$$

We have

(22) A1=1l2[4(∑t=1lt2−∑t=1(3/4)lt2)+14l3−4l∑t=(3/4)l+1lt]=1l(748l2+38l+16).

From the previous inequality this yields

(23) $$\begin{array}{c} {\left[R_{a}(l)\right]}^{2}\leq \frac{\left(l+2\right)\left(7l+4\right)}{12l^{2}} \end{array}$$

and the theorem follows.

### 3.3. Discussion

Theorems 2 and 3 have two meanings.

**Figure 2:**
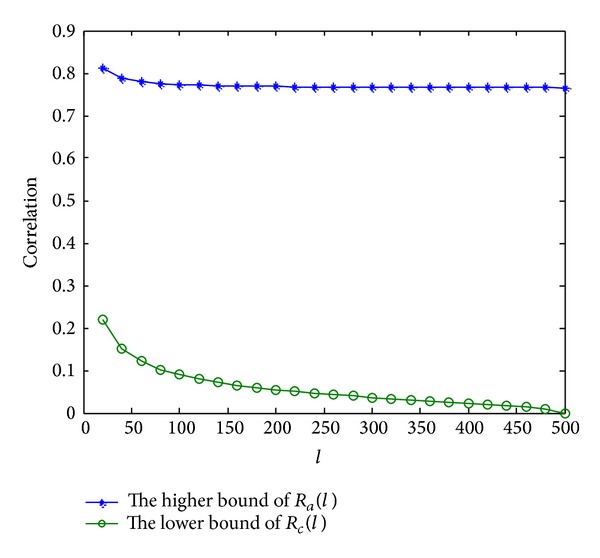
The higher bound of *R*_*a*_(*l*) and the lower bound of *R*_*c*_(*l*).

1. *R*_*c*_(*l*) cannot be arbitrarily small and *R*_*a*_(*l*) cannot be arbitrarily large.
2. *R*_*c*_(*l*) only contains the cross-correlation information and *R*_*a*_(*l*) only contains the autocorrelation information. Although they are more “pure” than *R*_max⁡_, generally they cannot be equal to 0 and 1, respectively. The lower bound of *R*_*c*_(*l*) and the higher bound of *R*_*a*_(*l*) indicate that there is some restrain relation between them.
3. The threshold to distinguish auto- and cross-correlation always exists.
4. Figure 2 gives the function graphs of the higher bound of *R*_*a*_(*l*) and the lower bound of *R*_*c*_(*l*). From it, we can see that no matter how small the cover is reserved, *R*_*a*_(*l*) is always larger than *R*_*c*_(*l*). That is to say the extractor can always find a threshold to distinguish auto- and cross-correlation in theory.

## 4. Conclusion

In this paper, we propose LAC&TCC properties and give the theoretical limits of SS watermarking sequences under the attacks of cropping and translation. From the perceptive of SS watermarking sequences, the future works include the following.

*(1) Considering Other Attacks. *In this paper, considering the complexity, we do not discuss rotation, scaling, and other attacks. Actually, these attacks often happen and are difficult to deal with. In the following research, we will add them to (1) and (2).

*(2) The LAC&TCC Sequences Design. *As described previously, the existing SS sequences do not have LAC&TCC properties and are not really fit for watermarking. It is necessary to design LAC&TCC sequences to improve performance of SS watermarking.
